# The effects of course format, sex, semester, and institution on student performance in an undergraduate animal science course

**DOI:** 10.1093/tas/txac004

**Published:** 2022-01-12

**Authors:** James R Vinyard, Francisco Peñagaricano, Antonio P Faciola

**Affiliations:** 1 Department of Animal Sciences, University of Florida, Gainesville, FL 32611, USA; 2 Department of Animal and Dairy Sciences, University of Wisconsin-Madison, Madison, WI 53706, USA

**Keywords:** animal nutrition, female performance, online learning, professor performance

## Abstract

The transition of courses from in-person to an online format due to the COVID-19 pandemic could have potentially affected overall student performance in lecture-based courses. The objective of this case study was to determine the impact of course format, as well as the effects of student sex, time of year at which the course was taken, and the institution it was taken at on student performance in an undergraduate animal science course. The course used for this study was taught at two institutions (University of Florida; UF and University of Nevada, Reno; UNR) over 7 yr (2014–2017 at UNR and 2018–2021 at UF). Student’s performance (*n* = 911) was evaluated using both quizzes and exams from 2014 through the spring semester 2020 and only exams were used for summer and fall semesters of 2020 and the spring and summer semesters of 2021. The final score (out of 100%) for each student was used to evaluate student’s performance. In addition, students were classified as high-performing students, if they scored ≥95% and low-performing students, if they scored ≤70%. The variables evaluated were the effects of semester (spring, summer, or fall), institution (UF or UNR), sex (male or female), number of teaching assistants (TAs; 0–13), and course format (online or in-person). The course was taught in-person at UNR and in-person and online at UF. The spring semester of 2020 was taught in-person until March but was switched to online approximately 9 wk after the semester started and was considered an online semester for this analysis. As the course was only taught online at UF, the variable course format was assessed using UF records only. Data were analyzed using both linear models and logistic regressions. The probability that students were high performing was not affected by sex or institution. Interestingly, both fall semester and the online format had a positive, desirable effect on the probability that students were high performing. The probability that students were low performing was not affected by sex. However, if a student performed poorly in the class, they were more likely to have taken the course at UNR, or at UF with many TAs. Thus, student’s performance was impacted by changing the course format, as well as institution, the number of TAs, and the semester in which the course was taken.

## INTRODUCTION

In March 2020, the world began to shut down due to the COVID-19 pandemic. The University of Florida (UF), along with most other universities in the United States, abruptly transitioned their courses to online formats and sent most undergraduate students home, which led to most courses being taught online for the remainder of the pandemic. This period, which lasted for over 18 mo, provided a unique opportunity to analyze student’s performance in courses that were traditionally offered in-person and had to be converted to an online format. The performance of undergraduate students in online courses is highly variable and largely dependent upon course structure and available course and student financial resources (Hansen and Reich, 2015), with students performing worse in courses with complex structures and better in courses when abundant resources are available (Jaggars and Xu, 2016). Online format may limit student–professor face-to-face interactions but may also provide a platform for introverted students to communicate via chat and other communication channels. Therefore, while some students may do better in in-person courses, others may benefit from online courses.

The factors that impact student success in university courses are multifaceted and one such factor is the institution at which a course is taught. University rank, which is based partly on academic rigor, could lead to differences in student’s performance as higher ranked institutions may be more likely to admit high-performing students. This would be further elucidated by differences in admissions criteria, like average grade point average (GPA), Scholastic Aptitude Test (SAT) score, or American College Testing (ACT) score, all of which have been reported to be good indicators of potential student performance (Schmitt et al., 2009). Thus, universities with higher admissions GPA and test scores would have high-performing students and universities with lower admissions GPA and test scores would have low-performing students.

Sex differences in academia have been of interest for decades; however, current literature indicate that there are no differences between males and females in science courses (Paul and Jefferson, 2019; Odom et al., 2021). While there may be no overall differences due to subject matter, there may be a difference in performance between sexes caused by the design of a specific course, as males generally do better with finite questions (multiple choice, true/false, etc.) and females generally do better with open-ended questions (essay or short answer; Tindall and Hamil, 2004). Thus, on a course-to-course basis, there may be differences in performance between sexes when there may not be on a course subject basis.

The quality of the professor, most frequently measured by end-of-semester reviews (Yunker and Sterner, 1988), can impact the performance of students as well. Meta-analytical review of literature indicates that the better reviews a professor receives at the end of the semester, the higher the test scores in the course are (Hedges et al., 1994). Carrell and West (2010) reported that the opposite can also be observed and when professors are rated more poorly, their students perform more poorly. However, ratings and performance may be confounded, as students may be more willing to rate a professor highly if they do well and poorly if they do poorly. Thus, there is interest in the effects of the rating of a professor on student’s performance.

The professor of the course, however, may not be the only source of instruction for students, as depending on the course they may have access to teaching assistants (TAs). According to Philipp et al. (2016), undergraduate students who had access to TAs in their courses performed higher than students who did not have access to them. However, TA instruction may not be equivalent to professor instruction (Weatherly et al., 2003) and the students may end up relying on their TAs rather than attending lecture or asking questions from the professor. Therefore, the impact of the number of TAs in each semester may influence student’s performance in a course.

Therefore, the objective of this case study was to determine the effect of course format, as well as student sex, time of year at which the course was taken, the number of TAs in a semester, and the institution it was taken at on student performance in an undergraduate animal science course.

## MATERIALS AND METHODS

According to the Institutional Review Board of the University of Florida guidelines, case studies, without student intervention, interaction, or identifiable private information, do not require approval.

### Course Information

Data for this analysis were collected using the final grades of students who took an undergraduate animal nutrition course (300 level, with similar prerequisites at both institutions) taught by the same professor across subsequent years. The professor’s prior teaching experience included 13 yr of teaching assistantship across four different courses: water buffalo production and digestion physiology (both at undergraduate and graduate levels) at the Federal University of Viçosa in Brazil; and ruminant nutrition (undergraduate) and ruminant nutrition physiology (graduate) at the University of Wisconsin-Madison. Other relevant prior experiences included formal TA training at UW-Madison, DELTA courses, and attending multiple teaching-related symposia and workshops.

Although lecture slides and exam questions had minor updates over the years, teaching material and exam questions were virtually the same across evaluated years. The course was taught at two different universities: University of Nevada at Reno (UNR) from 2014 to 2017 and the UF from 2018 to 2021. The instruction at UNR consisted of in-person lectures during spring semesters. The instruction at UF consisted of both in-person (2018 and 2019) and online (2020 and 2021) and was taught in the spring (2018–2021), summer (2019–2021), and fall (2020). In total, the course was taught over 12 semesters and 911 students completed the course. Data were not collected regarding race, ethnicity, age, sexuality, socioeconomic status, or gender identity. Student evaluations were done anonymously via university online platforms at the end of the semester; student response rates were in line with university response rates and ranged from 43% to 64%, at UF students are given in-class time to complete these evaluations, but they are not required. One problem is that students who greatly liked or disliked the course or instructor are more likely to respond and these may not reflect the overall student population. TAs were selected based on prior class performance and interview with instructor to assess interest and motivation. Responsibilities of TAs included attending lectures, holding weekly office hours, and help with grading and overall course management.

The semesters of instruction at UNR and the semesters at UF through the spring of 2020 consisted of weekly quizzes (best 10 out of 14) and exams (best 3 of 4) with an optional final that replaced the lowest exam grade. The course lecture was taught in-person until March 2020, when courses were switched to an online format due to the pandemic. The semesters from summer 2020 onward were taught using only exams (best 3 of 4) with an optional final that replaced the lowest exam grade as a source of evaluation. The spring semester of 2020, although it was taught partially in-person (exams 1 and 2) and partially online (exams 2, 3, and final), it was considered as an online semester for the purposes of this study as most of the points in the course were accrued online. All quizzes and exams were similar over the years, consisting primarily in short-answer questions with only a few questions that were either math based or multiple-choice/matching each semester. At both UF and UNR, the course was taught using Canvas (Instructure Inc., Salt Lake City, UT) as an online platform for notes and grades to be posted. Prior to the onset of the pandemic, quizzes and exams were administered on paper during lecture time and were supervised by the instructor and TAs. Once the course was moved online, quizzes were eliminated and exams were proctored via Canvas using the Honorlock (Honorlock Inc., Boca Raton, FL) extension to prevent cheating, exams went from 50 to 80 min to allow students to adjust to online format.

**Figure 1.**
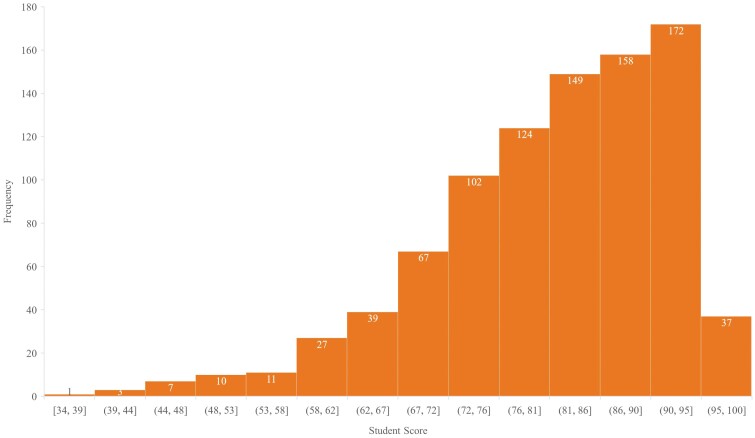
The distribution of student scores in a 300 level undergraduate animal science course taught by the same professor at both the UF and the UNR.

The variables that were considered were institution, sex, course format, semester, and number of TAs (Table 1). The variables by each semester the course was taught are shown in Table 2, along with the number of students in each semester, and the semester mean scores. The final score (out of 100%) for each student was used to evaluate student performance. In addition, students were classified as either high performing, if their grade was ≥95%, or low performing, if their grade was ≤70%. These two ranges were selected as the distribution of scores indicated (Figure 1) that most students scored between 70% and 95% in the course. Thus, those that would be either ≥95% or ≤70% would truly be considered high performing or low performing, respectively.

**Table 1.** The variables and their ranges investigated to determine their effects on student performance and the likelihood of students to be either high or low performing

Variable	Range
Institution	University of Nevada at Reno or University of Florida
Sex	Male or female
Semester^∗^	Spring, Summer, or Fall
Format^∗^	Online or in-person
Professor rating^†^	Score out of a 4.0 or 5.0 scale converted to a decimal
TA number^∗^	Ranging from 0 to 13 TAs per semester

This variable was only analyzed using the data from the UF, as the course was only taught in the spring and in-person with either no or one TA per semester at the UNR.

At the end of each semester, students rated the professor on a scale of either 1–4 (UNR) or 1–5 (UF) and an average score was provided and converted to a decimal to be used for the semester rating. Note that this variable is completely confounded with semester per academic year.

**Table 2.** Year, institution, course format, and professor rating each semester score in a level 300 undergraduate animal nutrition course taught by the same professor at both the UF and the UNR

Year	Semester	Institution^∗^	Format	Students			Score	Professor rating^†^	TA number^‡^
				Male	Female	Total	Mean ± SD		
2014	Spring	UNR	In-person	7	36	43	81.8 ± 10.8	0.89	0
2015	Spring	UNR	In-person	17	52	69	78.8 ± 11.8	0.83	1
2016	Spring	UNR	In-person	16	50	66	76.2 ± 11.6	0.78	1
2017	Spring	UNR	In-person	12	53	65	79.3 ± 10.4	0.90	0
2018	Spring	UF	In-person	25	105	130	83.1 ± 9.80	0.88	5
2019	Spring	UF	In-person	18	60	78	80.8 ± 10.1	0.91	6
	Summer	UF	In-person	9	13	22	83.9 ± 7.64	0.97	7
2020	Spring	UF	Online	17	85	102	86.6 ± 8.38	0.96	5
	Summer	UF	Online	9	51	60	82.1 ± 9.86	0.97	12
	Fall	UF	Online	19	108	127	80.9 ± 12.6	0.91	13
2021	Spring	UF	Online	16	82	98	81.8 ± 10.8	0.92	13
	Summer	UF	Online	7	44	51	78.8 ± 14.6	0.91	9

The institution at which the course was taught, either UNR or UF.

At the end of each semester, students rated the professor on a scale of either 1–4 (UNR) or 1–5 (UF) and an average score was provided and converted to a decimal to be used for the semester rating.

The number of TAs helping with the course in a given semester.

### Statistical Analysis: Effect of Institution and Sex

The effect of institution and sex on student performance was evaluated using the entire dataset (911 students). The following linear predictor was used


$$\begin{array}{l} \eta =\mu +\;institution\;+\;sex\;+\;semester\;(institution), \end{array}$$

where institution represents the fixed effect of institution (UNR or UF), sex represents the fixed effect of sex (female or male), and semester (institution) represents effect of the individual semester’s class. Final score was evaluated using the following linear model: re = η+ error, assuming that $error∼N\left(0,\sigma_{e}^{2}\right)$. In addition, both high and low performance were evaluated using the following logistic regression: log(P/[1-P])=   η where P represents the probability of either high performing (score ≥95%) or low performing (score ≤70%). All the analyses were performed on SAS.

### Statistical Analysis: Effect of Course Format, Semester, and Number of TAs

The effect of course format, semester, and number of TAs on student performance was evaluated using only the data collected from UF (668 students). Note that the course was taught in different semesters, both formats, and with multiple TAs only at UF. The following linear predictor was used


$$\begin{array}{l} \eta =\mu +format+semester+TAs, \end{array}$$

where format represents the fixed effect of course format (in-person or online), semester represents the fixed effect of course semester (spring, summer, or fall), and Tas represents the number of TAs assisting the course fitted as a linear covariate. Final score was evaluated using the following linear model: re=η+error, assuming again that $error∼N\left(0,\sigma_{e}^{2}\right)$. In addition, both high and low performance were evaluated using the following logistic regression: log(P/[1−P])= η, where *P* represents again the probability of either high performing (score ≥95%) or low performing (score ≤70%). All these analyses were also performed on SAS.

## RESULTS AND DISCUSSION

### Institution

The students who took the course at UNR received significantly lower final scores (*P* < 0.01) than the students who took the course at UF (Table 3). However, there were no differences (*P* ≥ 0.48) between UNR and UF on the likelihood of a student to be high performing or low performing (Table 3). The lower performance of students at UNR could potentially be due to differences in university rank between UNR and UF. Typically, universities that are higher ranked attract more competitive students, which could lead to higher performance at a higher ranked university (Jabjaimoh et al., 2019). Rankings are largely based on academic achievement and the US News and World Report Best Global University Rankings (USNWR) is a well-known ranking system that uses quantitative measurement for its ranking calculations but is highly correlated with the other ranking systems (Shehatta and Mahmood, 2016). In this instance, UF is ranked 5th as a public institution and UNR is ranked 112th (USNWR, 2021).

**Table 3.** The effects of institution, sex, course format, and semester on student score in a level 300 undergraduate animal nutrition course taught by the same professor at both the UF and the UNR

Variable	Mean (n)			SEM	P-value		
					F-test^∗^	High^†^	Low^‡^
	UNR		UF				
Institution	78.7 (243)		81.8 (668)	3.1	<0.01	0.77	0.48
	Male		Female				
Sex	79.7 (172)		80.8 (739)	1.7	0.16	0.53	0.37
	Online		In-person				
Format^||^	83.1 (438)		80.1 (230)	3.1	0.01	<0.01	0.49
	Spring	Summer	Fall				
Semester^||^	82.1 (408)^b^	80.9 (133)^b^	81.9 (127)^a^	4.0	0.55	0.03	0.61

These values are the results of an F-test to estimate differences in student performance due to each variable.

These values are the results of the likelihood of a student to be high performing (score ≥95%) due to the effect of each variable.

These values are the results of the likelihood of a student to be low performing (score ≤70%) due to the effect of each variable.

This variable was nested within institution when analyzed, as all potential levels were only possible at UF.

Means with different superscripts differ (*P* ≤ 0.05).

The undergraduate admissions criteria between UF and UNR may also explain as to why students received lower scores (worse performance) at UNR than UF. For admissions in 2021, UF applicants had an average GPA of 4.51, an average SAT score of 1,392, and an average ACT score of 31 (Lugo, 2021), resulting in an acceptance rate of 31% (USNWR, 2021), whereas for admissions in 2017 at UNR, applicants had an average GPA of 3.38, an average SAT score of 1,150, and an average ACT score of 24 (Patel, 2018), resulting in an acceptance rate of 87% in 2021 (USNWR, 2021). Thus, it is possible that students at UNR performed worse due to having lower rigor for their admissions, allowing for their distribution of students to have more low-performing students than UF.

Since students are as likely to be high performing at UF as they are at UNR, students who excel will excel regardless of the institution they are at. Similarly, since students are as likely at UF as they are at UNR to be low performing, students who are going to fail will fail regardless of the institution they attend. Thus, good students will be good students and bad students will be bad students regardless of where they take their courses, but the final scores the students will get depend somehow on the institution. However, as we only utilized data from students taking this specific course, this may not be able to be applied to all courses without further study.

### Sex

There was no effect (*P* ≥ 0.16) of sex on student performance neither on the likelihood of a student to be either high or low performing (Table 3). This is in corroboration with previous reports examining sex differences in student performance. Paul and Jefferson (2019) reported that, in an undergraduate environmental science course, there was no effect of sex on student performance neither in in-person nor in online sections. Moreover, a meta-analysis conducted by Odom et al. (2021) found that there are no differences in student performance due to sex in 169 different undergraduate natural science courses.

However, these results were not what we expected as we thought that female students may have been higher performing or more likely to be high performing. Tindall and Hamil (2004) discussed that females tend to outperform males in exercises in which practical knowledge can be applied and there is no distinct right or wrong choice. As the quizzes and exams in this course are predominantly short answer and essay questions with open-ended answers, they allow for full expression of knowledge rather than the sole memorization of facts (Pollina, 1995), which is why we expected that female students would outperform the male students. Typically, the top-performing students in this course were female, thus we expected to have that demonstrated in the results. However, 81% of the students that have taken this course have been female. Thus, with only 19% of the students in the course being male, it is reasonable to assume that since there were no differences between sexes for student performance, the majority of the top-performing students would be female.

### Semester

Students who took the course at UF in the fall had a greater likelihood to be high performing (*P* = 0.03) than those who took it either in summer or spring (Table 3). However, there was no effect of semester (*P* ≥ 0.55) on final score or the likelihood of students to be low performing (Table 3). Student performance between different types of semesters have differing results. Some reports indicate that students who take courses over the summer, when courses are accelerated to fit into a shorter time period, perform worse than students that take them during full length fall or spring semesters (Kretovics et al., 2005). Other reports indicate that students taking courses in the summer perform better due to decreased overall course loads, less course material, and smaller class sizes that allow for more student–professor interactions in the summer as compared with full fall or spring semesters (Allen et al., 1982; Lombardi et al., 1992; Fish and Kowalik, 2009; Fischer et al., 2020). However, most reports indicate that there are no differences between summer courses and standard spring or fall semester courses (Scott, 1995; Anatasi, 2007; Huntington-Klein and Gill, 2021). These reports corroborate our results of no difference in final score between spring, summer, and fall semesters and no difference in the likelihood of students to be low performing.

The increased likelihood of students to be high performing in the fall, compared with summer and spring could have also been caused by the economic status of their living situation while taking courses online (Attewell, 2001). Students from less wealthy families may have less ability to take classes in a quiet area that was more conducive to learning with a stable internet connection than students from wealthier families who may have better areas for learning and better internet availability (Hansen and Reich, 2015). Thus, when those students returned to campus in fall 2020, they would have had access to an environment that was better for learning and taking courses compared with semesters where students took courses from home. Therefore, economic disparities between students’ families may have caused the increased likelihood for students to be high performing while others perform worse due to the learning environment they have access to while taking courses online from home.

Another potential impact causing students in fall 2020 to be more likely to be high performing could be what is known as “Zoom fatigue” (Shoshan and Wehrt, 2021) that happened in the other semesters. Defined as the exhaustion caused by continuous virtual conferencing that has become more prevalent during the pandemic (Bennett et al., 2021), Zoom fatigue could be the reason for the differences in that semester. Fall 2020, although online, allowed students to return to their regular lifestyles, with the ability to be on campus, socialize with friends, and participate in some extracurricular activities. Zoom fatigue seems to be exacerbated by a lack of face-to-face contact in everyday life (Bennett et al., 2021; Shoshan and Wehrt, 2021), thus the partial return to normalcy could have improved the fatigued status of the students leading them to be more likely to be high performing.

### Format

There was no effect (*P* = 0.49) of course format (Table 3) on the likelihood for students to be low performing. However, students that took the course online received higher scores and were also more likely (*P* < 0.01) to be high performing than students that took the course in-person (Table 3). Although, the severity of the pandemic and its effects on daily life has been different every semester, these results would be the best indicators of the effects that the pandemic had on student performance, as all of the students who took the course online have had to take the course during the pandemic. However, as the course was not taught in-person during the pandemic, we are not able to fully separate the effects of the pandemic from the results of online students.

As mentioned previously, the reported impacts of the pandemic on student perception of stress and anxiety were varied. Most students reported increase in stress and decrease in mental health status (Wang et al., 2020). Previous reports have indicated that increase in overall stress levels and decreased mental health status can lead to decrease in student performance (Antaramian, 2014). While Wang et al. (2020) reported only a small fraction of students indicating lower stress during the pandemic, it seems as though perceived increases in stress did not negatively impact student performance in this case. While we did not evaluate stress levels or mental health status of students, decreased workload in the course with the absence of weekly quizzes and the cancelation of commitments outside their coursework and increased free time could have led to decreased stress due to the course (Wang et al., 2020). Studies evaluating mental health and student performance indicate that students with greater mental health statuses will typically be able to perform better in a course as compared with students of worse mental health status (Antaramian, 2014; Grøtan et al., 2019). Thus, this may explain why students that took the course online during the pandemic achieved higher scores and were more likely to be high performing than those that took the course in-person prior to the pandemic.

Another explanation for the greater likelihood for students to be high performing in an online setting is that students that may not participate in-person, may be more likely to participate online (Vonderwell and Zachariah, 2005). Typically, in online courses, it is reported that student participation decreases (Hrastinski, 2008). However, these studies are conducted primarily with prerecorded lectures rather than live, real-time lectures as in this course (Rothstein and Haar, 2020). While student participation was not recorded or measured, subjectively, students were more willing to participate during an online lecture in which they could type their questions into a chat box or ask them out loud than they were in-person when they could only ask them out loud. With previous studies (Rovai, 2000; Vonderwell and Zachariah, 2005) indicating that students who participate more in class perform better than those that do not participate or do not participate as much, the reason for students being more likely to be high performing online and perform better overall could possibly be increased participation. Not having weekly quizzes and having extra time for exams may also have helped some students to be high performing by decreasing their overall workload for the course.

### Semester per Academic Year

There was a significant effect of semester per academic year on final score, and also likelihood to be either high or low performing (*P* < 0.01). The nested effect of semester within institution captures the individual class effect, and as expected, there are differences between classes. Note that the class effect (semester per academic year), class size, and professor performance (average rating of the professor by the students) are confounded. Thus, changes in student performance from semester to semester can be related but not limited to the size of each class and the perceived performance of the professor (Table 2).

Changes in student performance have been reported in several studies across different institutions from primary education to the collegiate level when the professor was perceived by the students to be either high or low performing. Carrell and West (2010) reported that as students rated their professors at the United States Air Force Academy higher, the performance of those students was also higher. The same was stated for more poorly performing professors, in which their students performed worse. However, there is a correlation between the rating of the professor and the easiness of the course, in which students are more willing to rate their professors highly when they score well in the class and more willing to rate them low when they score poorly (Felton et al., 2003; Yining and Hoshower, 2003), which is partly indicated in Table 2 as most semesters with greater averages have higher ratings. However, ratings of professors are highly biased by other factors as well, which include but are not limited to low student response rates (Chapman and Joines, 2017), instructor sex (Mengel et al., 2018), and professors that are perceived as likable rating higher regardless of student learning (Hornstein, 2017), other factors may include professor appearance, course size, and difficulty. Thus, the semester-end evaluation of professors is not a reliable indicator of student performance, which led us to exclude it as a sole explanatory variable in our models, this is further evidence that professors’ evaluations must have a variety of input and not only student evaluations.

The effect of class size on student learning is frequently debated and is perceived to decrease student performance as the size of the class is increased at the university level. In a study of 760,000 undergraduates, Kokkelenberg et al. (2008) concluded that as university class sizes increased, the grades of the students decreased. While that may be true on a large scale, the study did not control for the instructor or course in that instance, as some courses may not be as impacted by class size. In the instance of this study, we found that the impact of class size is more variable and dependent on many factors, like fall 2020, larger classes can perform better than smaller classes. Overall, student performance is highly variable and only partially dependent on the size of the class, thus not allowing class size to be the sole predictor of performance (Ake-Little et al., 2020).

### Teaching Assistants

Student performance decreased (*P* < 0.01) as the number of TAs increased. Similarly, as the number of TAs increased, the likelihood of students to be high performing decreased (*P* = 0.01) and the likelihood of students to be low performing increased (*P* = 0.05). Philipp et al. (2016) reported that students who had access to TAs performed higher in undergraduate courses; however, in this instance, it seems that performance can be impaired by the use of too many TAs. The decreased student’s performance, decreased likelihood for the students to be high performing, and increased likelihood for the students to be low performing could be due to over-reliance on the TAs for instruction, rather than attending lecture and learning from the professor (Weatherly et al., 2003). However, Wheeler et al. (2016) indicated that student knowledge and performance were not reliant upon student use of TAs for the course; however, a very knowledgeable TA could improve both student confidence and performance in a course.

As mentioned previously, increased class sizes have the potential to cause decreased student performance as compared with smaller class sizes (Arias and Walker, 2004). In this course, the number of TAs recruited is typically related to the size of the class that semester, with larger classes having a higher number of TAs. Thus, the decreases in student performance with a greater number of TAs may be caused by the larger class size, rather than the number of TAs. However, we did not evaluate the performance of the TAs or the perception of TA performance by the students, thus it is difficult to discern whether negative impacts on student performance were caused solely by increased TA use.

### Limitations

The analysis of final scores reported residuals that were not perfectly normally distributed. During the preliminary analysis, transformation of student final scores and removal of outliers was attempted to normalize the distribution of the residuals (Lee and Nelder, 2001). However, due to the finite range of the response variable (0% to 100%), transformation of the data and removal of outliers were not possible as new outliers were continually identified after each removal and there was no identification of an ideal transformation (Breiman and Friedman, 1985). Anyway, we have decided to report the findings related to final scores because we believe that they still provide meaningful insight into the effect of course format, sex, semester, and institution on student performance.

Another limitation of this study is that it was not a controlled experiment and treatments were not applied to the student. This study was a unique case study, in which attempts were made to statistically control for the confounding variables. This study was also conducted only using the students taught by a single professor over multiple years, thus these results are only indicative of that cohort of students and require more data to be able to be extrapolated to a wide variety of courses or instructors. It is possible that teaching ability changes over the years; however, this is difficult to quantitatively assess and it has been minimized by the fact that the instructor had multiple years of previous teaching experience prior to start collecting these data.

## CONCLUSION

Students who took an animal nutrition course online at UF were more likely to be high performing than those who took the course in-person at UF potentially due to increased participation or decreased stress. Students showed worse performance, if they took the course with more TAs. Students who took the course at a smaller, lower ranked institution performed worse than those that took the course at a large, highly ranked institution. Interestingly, regardless of course format, institution, or semester, students were more likely to be high performing, when the professor was perceived to be better and more likely to be low performing, when the professor was perceived to be worse, indicating that student success or failure is tied to the performance of the professor, however, that can only currently be applied to the professor in this course. Finally, there was no greater likelihood for students to be high or low performing depending on their sex.
